# Heme acquisition in the parasitic filarial nematode *Brugia malayi*

**DOI:** 10.1096/fj.201600603R

**Published:** 2016-06-30

**Authors:** Ashley N. Luck, Xiaojing Yuan, Denis Voronin, Barton E. Slatko, Iqbal Hamza, Jeremy M. Foster

**Affiliations:** * New England BioLabs, Incorporated, Genome Biology Division, Ipswich, Massachusetts, USA;; † Department of Animal and Avian Sciences, University of Maryland, College Park, Maryland, USA;; ‡ Department of Cell Biology and Molecular Genetics, University of Maryland, College Park, Maryland, USA; and; § New York Blood Center, Lindsley F. Kimball Research Institute, New York, New York, USA

**Keywords:** iron metabolism, infectious disease, parasite metabolism

## Abstract

Nematodes lack a heme biosynthetic pathway and must acquire heme from exogenous sources. Given the indispensable role of heme, this auxotrophy may be exploited to develop drugs that interfere with heme uptake in parasites. Although multiple heme-responsive genes (HRGs) have been characterized within the free-living nematode *Caenorhabditis elegans*, we have undertaken the first study of heme transport in *Brugia malayi*, a causative agent of lymphatic filariasis. Through functional assays in yeast, as well as heme analog, RNAi, and transcriptomic experiments, we have shown that the heme transporter *B. malayi* HRG-1 (*Bm*HRG-1) is indeed functional in *B. malayi*. In addition, *Bm*HRG-1 localizes both to the endocytic compartments and cell membrane when expressed in yeast cells. Transcriptomic sequencing revealed that *Bm*HRG-1, *Bm*HRG-2, and *Bm*MRP-5 (all orthologs of HRGs in *C. elegans*) are down-regulated in heme-treated *B. malayi*, as compared to non–heme-treated control worms. Likely because of short gene lengths, multiple exons, other HRGs in *B. malayi* (*Bm*HRG-3–6) remain unidentified. Although the precise mechanisms of heme homeostasis in a nematode with the ability to acquire heme remains unknown, this study clearly demonstrates that the filarial nematode *B. malayi* is capable of transporting exogenous heme.—Luck, A. N., Yuan, X., Voronin, D., Slatko, B. E., Hamza, I., Foster, J. M. Heme acquisition in the parasitic filarial nematode *Brugia malayi.*

Human filarial nematode infections responsible for lymphatic filariasis (caused by *Wuchereria bancrofti*, *Brugia malayi*, and *Brugia timori*) and onchocerciasis (caused by *Onchocerca volvulus*) affect nearly 150 million people worldwide (1). Medical treatment relies on sustained mass drug administrations of microfilaricidal therapeutics (*e.g.,* albendazole, DEC, ivermectin) to disrupt transmission of the disease (2) but is contraindicated in regions where another filarial nematode, *Loa loa*, is endemic. Furthermore, as growing evidence of drug resistance in filarial nematodes emerges (3, 4), the development of safer macrofilaricidal treatment options has become an urgent need.

Although most cases of lymphatic filariasis are caused by *W. bancrofti*, *B. malayi* is frequently the subject of investigation because of its ability to maintain its life cycle in a laboratory setting. As with other filarial nematodes, transmission of *B. malayi* requires an arthropod vector (blood-feeding female mosquitoes) and a mammalian host (normally humans, although other mammals, *e.g.,* Mongolian jirds, are used in the laboratory). Within an infected mammalian host, *B. malayi* adult males and females reside in the lymphatic vessels, where they reproduce and release microfilariae (mf). The mf migrate to the capillaries from which they can be ingested by a mosquito during a blood meal. Within the insect vector, mf penetrate the midgut, enter the thoracic muscle cells, and remain intracellular for 2 molts before migrating *via* the hemolymph to the mouthparts of the mosquito.

Tetrapyrroles, such as heme, are used in every kingdom of life and have become indispensable to many biologic processes by serving as a cofactor for numerous proteins. Most organisms are readily able to synthesize heme (5); however, all nematodes (either free-living or parasitic) studied to date lack a complete and functional heme biosynthetic pathway (6). As heme auxotrophs, helminths must acquire heme from an exogenous source. Given the essential role of heme, this auxotrophy in nematodes may be exploited to develop drugs that interfere with heme uptake and utilization. Although *B. malayi* contains a functional ferrochelatase gene (the final step in the heme biosynthetic pathway and a likely product of lateral gene transfer from a Rhizobales-related species) (7), like other nematodes, *B. malayi* is incapable of synthesizing heme (6). However, unlike most nematodes, *B. malayi* (and most other filarial nematodes) contain *Wolbachia*, an obligate α-proteobacterial endosymbiont present within the lateral cords of male and female worms and in the developing oocytes and embryos in females that are required for worm fertility and development. Genomic sequencing of *Wolbachia* from *B. malayi* (*wBm*) revealed a complete and likely functional heme pathway (8). Certain trypanosomatids are also incapable of synthesizing heme, but contain a β-proteobacterial endosymbiont capable of synthesizing and supplying the vital cofactor (9, 10). Precisely how heme homeostasis is maintained in *B. malayi*, which may acquire heme from its environment, as well as perhaps from its *Wolbachia* endosymbiont, has remained an unanswered question.

Multiple heme responsive genes (HRGs) have been identified and assigned various functions within *Caenorhabditis elegans* (11–13). Paralogs *C. elegans* HRG-4 and -1 (*Ce*HRG-4 and -1) (11), are both high affinity regulated heme transporters involved in uptake and trafficking of heme within the intestine of *C. elegans*. Two other *Ce*HRG-4 paralogs, *Ce*HRG-5 and -6, are also involved in heme uptake within the intestine (13). Although heme uptake into the intestine is redundant (*Ce*HRG-4–6 are all involved at various heme concentrations), heme export from the intestine is accomplished *via* the ABC-transporter *C. elegans* multidrug resistance protein 5 (*Ce*MRP-5) (14). In addition, *Ce*HRG-2 (a type I transmembrane protein involved in heme uptake and utilization within the hypodermis) (15) and *Ce*HRG-3 (likely a heme chaperone involved in delivering maternal heme to developing oocytes) (12) have both been well characterized in *C. elegans*.

The *B. malayi* orthologs of *Ce*HRG-1 [*B. malayi* HRG-1 (*Bm*HRG-1), Bm5182, WormBase ID: WBGene00225443], *Ce*HRG-2 (*Bm*HRG-2, Bm2383, WormBase ID: WBGene00222644), and *Ce*MRP-5 [*B. malayi* multidrug resistance protein 5 (*Bm*MRP-5, Bm3373, WormBase ID: WBGene00223634] have all been identified, on the basis of protein sequence homology. However, probably because of short gene lengths that are split into multiple exons and large gaps in the genome assembly, *Bm*HRG-3–6 have not been identified. Despite this, and to further understand the biology of heme metabolism in filarial worms, a potential target for filariasis control, we have undertaken this first study of heme homeostasis in *B. malayi.*

## MATERIALS AND METHODS

### Yeast strains and growth medium

The *Saccharomyces cerevisiae* strains used in this study were derived from the W303 and YPH499 backgrounds. The *hem1*Δ(6D) and OPY102 strains were constructed as described elsewhere (16, 17). To construct *hem1*Δ *fre1*Δ *fre2*Δ MET3-FRE1, plasmid pRS404-MET3-FRE1 was linearized with *Nde*I and integrated into the TRP1 locus of OPY102. Cells were maintained in yeast peptone dextrose (YPD) or appropriate synthetic complete (SC) medium supplemented with 250 μM δ-aminolevulinic acid (ALA) (Frontier Scientific, Inc., Logan, UT, USA) (18).

A codon optimized *Bm*HRG-1 ORF (±HA tag) for yeast expression was synthesized by IDT (Integrated DNA Technology, Coralville, IA, USA), amplified by PCR using gene-specific primers containing the *Bam*HI and *Xba*I sites, digested, and ligated to pYES-DEST52 vector (Thermo Fisher Scientific Life Sciences, Carlsbad, CA, USA) digested with the same enzymes.

### Spot growth assay

The *hem1Δ S. cerevisiae* yeast strain lacks the first enzyme in the heme biosynthetic pathway, ALA synthase (ALAS). Because of the lack of ALAS, ALA (the product of ALAS) or excess hemin must be supplied exogenously in the growth medium, for the *hem1Δ* strain to grow. Plasmids for *Bm*HRG-1 expression were transformed into strain *hem1*Δ(6D) using the lithium acetate method (19). Transformants were selected on 2% w/v glucose SC-Ura plates supplemented with 250 μM ALA. Five or 6 transformed colonies were picked and streaked on 2% w/v raffinose SC-Ura plates supplemented with 250 μM ALA for 48 h to deplete glucose. Before spotting, the cells were cultivated in 2% w/v raffinose SC-Ura medium for 18 h to deplete hemin. Cells were then suspended in water to an OD_600_ of 0.2. Ten-fold serial dilutions of each transformant were spotted (10 μl/spot) onto 2% w/v raffinose SC-Ura plates supplemented with 0.4% w/v glucose and 250 μM ALA (positive control) or 0.4% w/v galactose and different concentrations of hemin and then incubated at 30°C for 3 d before imaging.

### Ferrireductase assay

The strain *hem1*Δ *fre1Δ fre2Δ* MET3-FRE1 was used for the ferrireductase assay. The iron- and copper-regulated endogenous genes for *FRE1* and *FRE2* (20, 21) have been deleted in this strain, which instead contains only 1 ferric reductase (FRE1) under control of the inducible MET3 promoter, thus making it possible to directly assay any changes in intracellular heme *via* ferric reductase activity caused by the expression of HRG-1 (22). Yeast transformation and selection were performed as described above using respective SC auxotrophic medium supplemented with 250 μM ALA. After being depleted of hemin in 2% w/v raffinose SC-Ura, -Trp, -Met medium for 12 h, cells were suspended in 2% w/v raffinose SC-Ura, -Trp medium supplemented with 0.4% w/v galactose, 0.1 mM Na_2_S, and various concentrations of hemin to an OD_600_ of 0.3. These were cultivated in 96-well plates at 30°C, with shaking at 225 rpm for 16 h and assayed for ferrireductase activity (20). The cells were washed with washing buffer (2% bovine serum albumin, 0.1% Tween-20 in 2× PBS) 3 to 4 times to remove residual hemin in the medium, washed twice with reaction buffer [(5% glucose and 0.05 M sodium citrate buffer (pH 6.5)], suspended in reaction buffer and the OD_600_ determined using a plate reader. Equal volume of assay buffer (2 mM bathophenanthroline disulfonate, 2 mM FeCl_3_ in reaction buffer) was added to the cells (*t* = 0 min) and incubated at 30°C in the dark until red color developed. OD_535_ and OD_610_ were determined, and ferrireductase activity (nmol/10^6^cells/min) was calculated as:


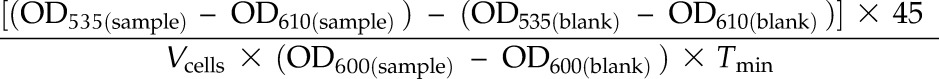


### β-Galactosidase reporter assay

The plasmids for *Bm*HRG-1 expression were cotransformed into strain *hem1*Δ(6D) with pCYC1-LacZ. Selection of transformants was performed as described above using appropriate SC auxotrophic medium supplemented with 250 μM ALA. Cells were depleted of hemin in 2% w/v raffinose SC-Ura, -Trp medium for 12 h, and then were suspended in 10 ml 2% w/v raffinose SC-Ura, -Trp medium supplemented with 0.4% w/v galactose, and different concentrations of hemin to an OD_600_ of 0.1. Cells were cultivated at 30°C, with shaking at 225 rpm for 12 h and assayed for β-galactosidase activity, as described elsewhere (23). β-Galactosidase activities were normalized to total protein concentration.

### Immunoblot analysis

For Western blot analysis experiments, yeast transformants were resuspended in lysis buffer [1% SDS, 8 M urea, 10 mM Tris-HCl (pH 8.0), and 10 mM EDTA] with protease inhibitors (1 mM PMSF, 4 mM benzamidine, 2 μg/ml leupeptin, and 1 μg/ml pepstatin) and 0.5 mm diameter acid-washed glass beads. Cells were heated at 65°C for 10 min and disrupted in FastPrep-24 (MP Biomedicals, Santa Ana, CA, USA) 3 times for 30 s each at the 6.5 m/s setting. Cell lysates were collected, and the total protein concentration was quantified with the Bradford reagent (Bio-Rad, Hercules, CA, USA). Protein samples were resolved on 12% SDS-polyacrylamide gel and transferred to nitrocellulose membrane (Bio-Rad). For immunoblot analysis, the membranes were incubated with rabbit anti-HA (Sigma-Aldrich, St. Louis, MO, USA) as primary antibody at a 1:5,000 dilution for 16 h at 4°C, followed by HRP-conjugated goat anti-rabbit antibody at a 1:10,000 dilution for 1 h at room temperature. Signal was detected using SuperSignal chemiluminescence reagents (Thermo Fisher Scientific Life Sciences) in a gel documentation system (Bio-Rad).

### Immunofluorescence

Yeast transformants were cultivated in 2% w/v raffinose SC-Ura medium supplemented with 0.4% w/v galactose and 250 μM ALA to midlog phase and then fixed with 4% formaldehyde for 1 h at room temperature. Immunofluorescence microscopy was performed as described elsewhere (23). Images were taken using a DM IRE2 epifluorescence microscope (Leica, Wetzlar, Germany) connected to a Retiga 1300 cooled Mono 12-bit camera (Retiga, QImaging, Surry, BC, Canada).

### *B. malayi in vitro* culture

Unless otherwise noted, *B. malayi* mf and adult worms (TRS Labs, Athens, GA, USA) were incubated in RPMI 1640 medium (containing 25 mM HEPES, 5 mM glutamine, 200 μg/ml penicillin, and 200 μg/ml streptomycin) at 37°C, 5% CO_2_. All hemin and heme analog solutions were prepared in 300 mM ammonium hydroxide and pH adjusted to pH 8.0 with 6 M HCl before filter sterilization.

### Production of rabbit polyclonal antibodies to *Bm*HRG-1

Anti-*Bm*HRG-1 serum was raised against a peptide of the 18 C-terminal residues of *Bm*HRG-1 conjugated to KLH *via* an N-terminal cysteine using *m*-maleimidobenzoyl-*N*-hydroxysuccinimide ester (MBS; Thermo Fisher Scientific Life Sciences) (24). Sera were raised in rabbits by Covance Immunology Services (Princeton, NJ, USA). Antibodies were purified according to a published procedure (25).

### *B. malayi* protein extraction and immunoblot analysis

Live *B. malayi* mf and adult male and female worms were incubated for 24 h in RPMI-1640 containing 0 (control), 5, 20, or 100 μM hemin chloride (Frontier Scientific, Inc.) before being flash frozen at −80°C. For extraction of total protein, frozen worm samples were thawed on ice before being washed 3 times with 200 μl of 1× PBS (pH 7.4). Samples were resuspended in 200 μl of tissue extraction reagent I (Thermo Fisher Scientific Life Sciences) containing protease cocktail inhibitor (Sigma-Aldrich). Worm samples were then homogenized with ceramic beads in CK14 tubes (3 × 30-s 5000 rpm pulses, with 1 min ice in between) using a Minilys homogenizer (Precellys-Bertin Technologies, Rockville, MD, USA). Protein concentrations were determined with Bradford reagent (Bio-Rad) (26). Protein extracts were resolved by SDS-PAGE on 10–20% Tris-glycine gels (Thermo Fisher Scientific Life Sciences). Proteins were transferred to nitrocellulose (200 mA, constant for 2 h). Following overnight blocking in 5% milk/ Tris-buffered saline/0.1% Tween-20 (TBST), the immunoblot was analyzed using the purified anti-*Bm*HRG-1 rabbit sera described above (1:5000 dilution in 2% milk/TBST) as the primary antibody. A horseradish peroxidase-linked anti-rabbit secondary antibody (Cell Signaling Technology, Danvers, MA, USA) and the ECL Western Blot analysis Detection Kit (GE Healthcare, Pittsburgh, PA, USA) were used to detect bound antibody.

### Measurement of intracellular *B. malayi* heme concentration

The heme concentrations within *B. malayi* adults and mf were determined by using a published pyridine hemochrome method (27, 28). In brief, adult worms (18 females and 27 males) were washed once in PBS. For mf studies, to normalize each sample by number of mf, a 1:100 dilution of each mf sample (1 ml) was counted before centrifugation and a subsequent wash in PBS. All samples were then stored at −80°C until further use. For heme extraction and quantification, the samples were resuspended in 840 μl of 1 mM Tris-HCl (pH 8.0) and homogenized with ceramic beads in CK14 tubes using a Minilys homogenizer (Precellys-Bertin Corp.) (30-s 5000 rpm pulses 3 times, with 1 min ice in between). Another 840 μl of 1 mM Tris-HCl (pH 8.0) was added, and mixed (total volume 1680 μl). After 840 μl was transferred into each of two 13 × 100 mm glass tubes (duplicates), 100 μl of 1 N NaOH was added, and the tube vortexed. After 2 min, 200 μl of pyridine solution (Sigma-Aldrich) was added and mixed by vortexing. Samples were transferred to a 1 ml cuvette, and the baseline absorbance (at 541 and 557 nm) was read. Sodium hydrosulfite (Sigma-Aldrich) crystals (2–3 mg) were added, the sample was mixed by gentle pipetting and the reduced absorbance at 541 and 557 nm acquired. Heme concentrations were calculated based on millimolar absorption coefficient of 20.7 for the difference in absorption between the spectrum peak at 557 nm and the valley at 541 nm. *B. malayi* mf heme concentrations were normalized based on mf counts in each sample.

### *Ex vivo* motility of heme-treated *B. malayi*

*B. malayi* adults were cultured (1 male or female/well, in 24-well plates) in 1 ml RPMI-1640 medium (control), supplemented with 5, 20, or 100 μM hemin chloride at 37°C. Medium was changed every 2 d. Motility was scored daily, based on a previously described motility scoring system (7), where 0 is nonmotile and 4 is highly active.

### Gallium protoporphyrin IX toxicity assays

*B. malayi* adults were cultured (6 adult male or female worms/group) in RPMI-1640 medium supplemented with either gallium protoporphyrin IX (GaPPIX; Frontier Scientific, Inc.) or gallium chloride (GaCl_3_; Sigma-Aldrich). The effect of heme on GaPPIX cytotoxicity was examined by incubating *B. malayi* adults with 2, 5, or 20 μM GaPPIX and increasing hemin concentrations (10, 20, or 50 μM heme). Motility was assayed as described above. Viability of GaCl_3_-treated worms was determined by the previously described MTT assay (29, 30).

### Zinc mesoporphyrin pulse-chase analysis

*B. malayi* adults were cultured (1 male or female/well, in 24-well plates) in RPMI-1640 medium containing 40 μM zinc mesoporphyrin (ZnMP; Frontier Scientific, Inc.) for 18 h at 37°C (previously determined conditions, data not presented). Fluorescently labeled worms were then transferred into fresh RPMI-1640 medium (1 adult male or female/well, in 24-well plates) containing unlabeled hemin chloride. Control worms were transferred into fresh RPMI-1640 medium containing no heme analog. At timed intervals (0, 2, 4, 8, and 24 h), aliquots of worms (8 adult male and female worms/timepoint) were fixed in 80% ethanol, mounted on a slide, and immediately analyzed using an epifluorescence microscope [differential interference contrast (DIC) and rhodamine channels; Axiovert 200M; Zeiss, Oberkochen, Germany].

### *Bm*HRG-1 hairpin small interfering RNA preparation

The preparation of *Bm*HRG-1 hairpin small interfering (hsi)RNA was as previously described by Landmann *et al.* (31). Total RNA (700 ng from *B. malayi* females) was used as a template for the production of cDNA, with random primers and the ProtoScript AMV First Strand cDNA Synthesis Kit (New England BioLabs, Inc.). DNA templates for *in vitro* transcription were generated by PCR using Crimson Taq DNA Polymerase (New England BioLabs, Inc.) and *Bm*HRG-1 gene–specific PCR primers (designed to yield a PCR product corresponding to ∼400 bp and also containing a T7 promoter sequence followed by 2 guanine bases at the 5′ end for transcription by T7 RNA polymerase; left primer: 5′-TAATACGACTCACTATAGGGGGCTTTGACATGCAAGATGA-3′, right primer: 5′-TAATACGACTCACTATAGGGATACCACGCCGAAAGCATAG-3′) (Integrated DNA Technology). *Bm*HRG-1 specific double-stranded (ds)RNA was prepared using the T7 Quick High Yield RNA Synthesis Kit (New England BioLabs, Inc.) and purified by isopropanol precipitation. The dsRNA was processed into hsiRNA using ShortCut RNase III (New England BioLabs, Inc.), purified by ethanol precipitation and resuspended in distilled H_2_O. Agarose gel electrophoresis alongside the siRNA Marker (New England BioLabs, Inc.) was used to examine the size and purity of the *Bm*HRG-1 hsiRNA. A Nanodrop spectrophotometer (Thermo Fisher Scientific Life Sciences) was used to quantify *Bm*HRG-1 hsiRNA.

### ***I****n vitro* hsiRNA soaking and analysis of *Bm*HRG-1 knockdown

*In vitro* RNA interference was accomplished by adding various amounts of *Bm*HRG-1 hsiRNA (0–5 μM) to a 12-well plate and adding warm RPMI-1640 medium (1 ml final volume). Adult female worms (1 worm/well, 6 worms/treatment group) were incubated at 37°C (5% CO_2_), and the hsiRNA/medium was changed every 12 h. Microfilarial output from female worms was assessed after every hsiRNA treatment by counting the number of mf in 10 μl of medium from each well.

*Bm*HRG-1 knockdown was assessed by using various experimental methods: *1*) Following 48 h (4 total treatments) of hsiRNA (0, 1 or 5 μM), worms were soaked in RPMI-1640 medium containing 40 μM ZnMP for 18 h before the fluorescence was visualized, as described above. *2*) After 24 h of soaking in 0.5 μM *Bm*HRG-1 hsiRNA, worms were soaked in RPMI-1640 containing 20 μM heme (with or without RNAi) for 7 d (medium was changed every 24 h before heme was extracted, and total intracellular heme content determined. *3*) After 24 h of soaking in *Bm*HRG-1 hsiRNA (0.5 or 1 μM), worms were soaked in RPMI-1640 containing GaPPIX (0, 1, 2, 5, or 20 μM, with or without RNAi). Medium was changed every 24 h, and motility was scored daily as described above. Control worms were treated in a similar manner in the absence of any hsiRNA.

### Transcriptome sequencing and bioinformatic analysis

*B. malayi* mf and adult worms (1 male or female/well, in 24-well plates) were incubated in RPMI 1640 medium (control) supplemented with 20 or 100 μM heme (hemin chloride) at 37°C . After 24 h, the worms were harvested, flash frozen in liquid nitrogen and stored at −80°C until further use. For RNA preparation, samples were homogenized with ceramic beads in CK14 tubes using a Minilys homogenizer (as above) and total RNA was extracted by organic extraction with Trizol (Thermo Fisher Scientific Life Sciences). Samples were treated with DNase I (Thermo Fisher Scientific Life Sciences) before further Trizol extraction and final purification. The RNA integrity, purity and concentration of all samples were assessed with a Bioanalyzer 2100 (Agilent Technologies, Lexington, MA, USA). Samples were prepared for sequencing by using the NEBNext mRNA Library Prep Master Mix Set for Illumina (New England BioLabs, Inc.). Library quality was assessed before transcriptomic sequencing of 50 bp single-end reads on an Illumina Genome Analyzer IIx sequencer.

All data was analyzed with a local version of Galaxy (32–34). Sequence reads from each tissue sample were first assessed for quality using FastQC (v., 1.0.0; Babraham Institute, Babraham, United Kingdom; *http://www.bioinformatics.babraham.ac.uk/projects/fastqc/*) (35), and further analyzed by using the Tuxedo protocol (36). RNA-Seq reads from each sample were aligned to the *B. malayi* genome (Wormbase, v. WS236) using TopHat, (v. 1.4.1; Johns Hopkins University, Baltimore, MD, USA; *http://ccb.jhu.edu/software/tophat/index.shtml* (37). Default parameters were used, except that the maximum number of alignments allowed was set to 40. Reads aligned using TopHat were assembled into transcripts by using Cufflinks (v. 1.3.0; Cole Laboratory, University of Washington, Seattle, WA, USA; *http://cole-trapnell-lab.github.io/cufflinks/*). Default parameters were used. Cufflinks assemblies from all samples were merged by Cuffmerge (v.1.0.0) and used for differential expression testing by Cuffdiff (v.1.3.0), with the false-discovery rate set to 0.01.

### *In situ* hybridization

*B. malayi* cDNA was synthesized from 1 μg of adult female total RNA using the Protoscript II First Strand cDNA Synthesis Kit (New England BioLabs, Inc.). *Bm*HRG-1 primers were designed with Primer 3 and synthesized by Integrated DNA Technology. *Bm*HRG-1 was amplified by PCR using Taq DNA polymerase (New England BioLabs, Inc.) from the *B. malayi* 1st strand cDNA. Amplified *Bm*HRG-1 fragments were ligated into the pCRII vector (Thermo Fisher Scientific Life Sciences). Ligated plasmids were transformed into INVαF′ competent cells (Thermo Fisher Scientific Life Sciences). Transformed cells were grown on X-Gal/IPTG plates, the recombinants selected and grown overnight before plasmid isolations, using the Monarch Plasmid Miniprep Kit (New England BioLabs, Inc.). *Bm*HRG-1 inserts were confirmed by sequencing and used as templates for RNA probe labeling.

Sense (negative control) and antisense (experimental) RNA probes were prepared by *in vitro* transcription from linearized plasmids (1.25 μg) containing *Bm*HRG-1 inserts with flanking Sp6 and T7 RNA polymerase transcription start sites. In brief, reactions (60 μl) contained 1× RNA polymerase buffer (New England BioLabs, Inc.); 20 nmol each of dCTP, dGTP, dATP, and fluorescein-12-dUTP (FITC-dUTP; Thermo Fisher Scientific Life Sciences); 40 units RNase Inhibitor (New England BioLabs, Inc.); and 80 units of RNA polymerase (Sp6 or T7; New England BioLabs, Inc.) for 4 h at 40°C. Synthesized RNA probes were DNAse I (Thermo Fisher Scientific, Inc.) treated (37°C for 30 min), purified by ethanol precipitation, resuspended in 1× TE buffer, and stored at −80°C.

For the visualization of the RNA probe, *B. malayi* adult females were fixed and permeabilized with 4% formaldehyde in PBS (Sigma-Aldrich) with 0.1% Triton-X100 for 20 min. During fixation, worms were cut several times to improve the penetration of the reagents. Samples were then washed 3 times in PBS. Additional permeabilization of samples was performed by incubating with Proteinase K [20 µg/ml in 50 mM Tris-HCl, (pH 7.4); New England BioLabs, Inc.] 15 min at 37°C. The samples were washed with PBS and prehybridized for 2 h at 58°C in hybridization buffer [45% deionized formamide, 4× saline sodium citrate (SSC) buffer, 10 mM DTT, 100 µg/ml yeast transfer RNA, and 40 µg/ml denatured and sheared salmon sperm DNA). The buffer was replaced with fresh hybridization buffer containing 10 ng of the probe, and the samples were incubated at 58°C overnight. After the hybridization, samples were washed 2 times (15 min each) with 2× SSC buffer at 37°C, 2 times (15 min each) with 1× SSC buffer at 37°C, and 2 times (15 min each) with 0.5× SSC buffer at room temperature. To digest unbound single-stranded RNA probe, samples were incubated with RNase A [20 µg/ml, in 500 mM NaCl, 10 mM Tris-HCl, 1 mM EDTA (pH 8.0)] at 37°C for 1 h. The samples were washed in PBS and mounted in glycerol/PBS (1:1) solution on a slide. The samples on the slides were viewed using a 510 Meta confocal microscope (Zeiss, Oberkochen, Germany). The same procedure was performed for both sense- and antisense-oriented RNA probes.

## RESULTS

### *Bm*HRGs

**Figure 1.**
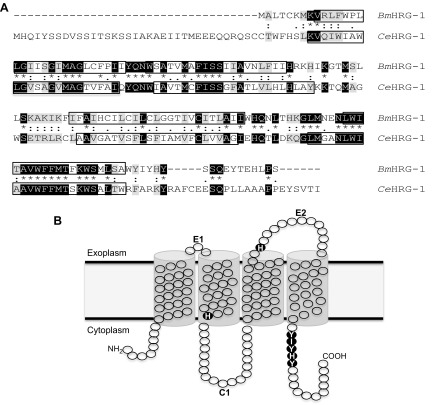
*A*) ClustalW amino acid alignment shows similarities between *Bm*HRG-1 and *Ce*HRG-1. Identical residues are shown in black, while conserved residues are in gray. *B*) The putative membrane topology of *Bm*HRG-1 includes 4 transmembrane domains (boxed regions in *A*, as predicted by TMHMM 2.0) as well as a conserved histidine residue (His100) and C-terminal motif (YIYHY, dark circles) important for heme uptake (38).

To date, only the *B. malayi* orthologs of *Ce*HRG-1 (*Bm*HRG-1), *Ce*HRG-2 (*Bm*HRG-2), and *Ce*MRP-5 (*Bm*MRP-5) have been identified based on sequence homology. *Bm*HRG-1, although slightly smaller in size (148 *vs.* 194 aa), was identified because of its sequence homology to *Ce*HRG-1 (∼39% identical) (**Fig. 1*A***). The *Bm*HRG-1 (also known as *Bm*5182, WormBase ID: WBGene00225443) locus (*Bm*al_v3_scaffold110:17681-19284) is composed of 4 exons. As with *Ce*HRG-1, *Bm*HRG-1 is predicted to contain 4 transmembrane helices connected by 2 exoplasmic loops, whereas the N and C termini are predicted to be cytoplasmic (Fig. 1*B*; TMHMM, v. 2.0; Prediction of Transmembrane Helices in Proteins; Center for Biological Sequence Analysis, Technical University of Denmark, Lyngby, Denmark; *http://www.cbs.dtu.dk/services/TMHMM/*). Two histidine residues, one in extracellular loop 2 and another in the second transmembrane domain (His90/His135 in *Ce*HRG-1) identified as being critical to heme transport, are conserved in *Bm*HRG-1 (His55/His100) (38). Moreover, a C-terminal cluster of aromatic and basic amino acids (FARKY in *Ce*HRG-1) (38), which may be involved in translocation of heme into the cytoplasm, is conserved in *Bm*HRG-1 (YIYHY).

HRG-2, through experiments in *C. elegans*, has been shown to be a slightly different member of the HRG family. A single-pass type I transmembrane protein, *Ce*HRG-2 localizes to the endoplasmic reticulum and apical plasma membrane of hypodermal cells (15). As a single-pass membrane protein, *Ce*HRG-2 is unlikely to function as a heme transporter, but contains a thioredoxin-like fold and glutathione *S*-transferase domain in the C terminus that may allow it to function as an oxidoreductase (13). Although the 2 are fairly similar (∼31% identical/51% similar), *Bm*HRG-2 is slightly larger in size (290 *vs.* 279 aa) than its *C. elegans* homolog and does not contain any predicted transmembrane domains. However, *Bm*HRG-2 does maintain the thioredoxin-like fold and glutathione-*S* transferase domain found in *Ce*HRG-2 and may also therefore serve as an oxidoreductase, although its intracellular location remains unclear.

In *C. elegans*, an ABC transporter, *Ce*MRP-5, localizes to the basolateral membrane of the intestine and appears to be the only mechanism for heme export from the intestine of *C. elegans* (14). The *B. malayi* homolog, *Bm*MRP-5, is fairly similar (48% identical/66% similar) to *Ce*MRP-5 (1473 *vs.* 1400 aa) and is likely to function in much the same manner.

### *Bm*HRG-1 overexpression, localization, and *in vivo* functionality

**Figure 2.**
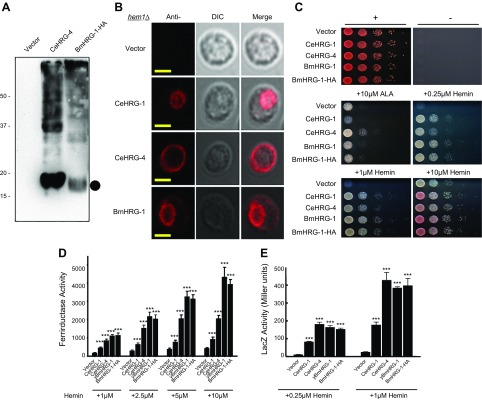
*Bm*HRG-1 overexpression, localization, and functionality in *S. cerevisiae.* *A*) Immunoblot analysis of yeast transformants overexpressing C-terminal HA-tagged versions of *Ce*HRG-4 (5 μg total extract) or *Bm*HRG-1 (30 μg total extract) in the *hem1*Δ strain. The predicted molecular mass (∼17 kDa) of *Bm*HRG-1 is indicated by the filled circle. *B*) Indirect immunofluorescence images of the *hem1Δ* strain transformed with vector alone, *Ce*HRG-1-HA, *Ce*HRG-4-HA, or *Bm*HRG-1-HA. Scale bars, 5 μm. *C*) Spot-growth assay. Yeast (*hem1Δ*) transformed with empty vector, *Ce*HRG-4, *Ce*HRG-1, *Bm*HRG-1, or *Bm*HRG-1-HA spotted in serial dilutions on plates supplemented with 10 μM ALA or the indicated hemin concentration and incubated at 30°C for 3 d before imaging. Positive control (top left): +0.4% glucose, +250 μM ALA; negative control (top right): −ALA, −hemin. *D*) The *hem1Δfre1Δfre2Δ*MET3-FRE1 strain was transformed with empty vector, *Ce*HRG-1, *Ce*HRG-4, *Bm*HRG-1, or *Bm*HRG-1-HA and ferric reductase activity (nanomoles/10^6^cells/min) was measured in the presence of hemin (as indicated). *E*) The *hem1Δ* strain stably expressing either the pYes-DEST52 vector alone, *Ce*HRG-1, *Ce*HRG-4, or *Bm*HRG-1 (untagged as well as HA tagged) were transformed with pCYC1-LacZ. Following growth in the indicated concentrations of hemin after 12 h, β-galactosidase activity of the cell lysates was determined. The results are means ± sem of 3 independent experiments. ***P < 0.001, Student’s *t* test.

Immunoblot analysis of lysates from the yeast *hem1Δ* strain expressing a codon-optimized C-terminal HA-tagged *Bm*HRG-1 revealed that *Bm*HRG-1 migrates at the predicted molecular mass (∼17 kDa; **Fig. 2*A***). However, expression of *Bm*HRG-1 was much lower than the *Ce*HRG-1 paralog *Ce*HRG-4. As with overexpression of other HRGs in yeast, multiple oligomeric forms of *Bm*HRG-1 are detected. Although *Ce*HRG-1-HA appears to localize to endosomal compartments (yeast vacuole), and *Ce*HRG-4-HA localizes to the plasma membrane, *Bm*HRG-1-HA appears to localize to both membranes, as indicated by indirect immunofluorescence of the transformed hem1Δ strain (Fig. 2*B*).

To verify the functionality of *Bm*HRG-1, spot growth assays were performed using the *hem1Δ S. cerevisiae* yeast strain, growth of which necessitates that either ALA or hemin be exogenously supplied (Fig. 2*C*). As with *Ce*HRG-1 and -4, we found that yeast transformed with *Bm*HRG-1 (with or without the HA-tag) rescued growth of the *hem1*Δ strain in relatively low concentrations of hemin (0.25 *vs.* 10 μM for the vector control).

Using the transformed *hem1*Δ yeast strain, we assayed changes in the intracellular heme pool as a result of expressing *Bm*HRG-1. First, we measured the ferrireductase activity (a heme-dependent enzyme) in the *hem1Δfre1Δfre2Δ*MET3-FRE1 strain transformed with *Ce*HRG-1, *Ce*HRG-4, *Bm*HRG-1, or *Bm*HRG-1-HA. Heme imported by *Bm*HRG-1 was indeed incorporated into intracellular hemoproteins, as evidenced by a significant increase in ferrireductase activity (Fig. 2*D*). Next we measured intracellular heme as a function of Hap1-5 regulated β-galactosidase activity from a *CYC1*::*lacZ* promoter–reporter fusion. In this experiment, β-galactosidase activity is a direct measure of intracellular heme content, as *lacZ* expression is dependent on Hap1-5, a heme-binding transcription factor (38). As with *Ce*HRG-1 or -4, the *hem1*Δ yeast strain expressing the CYC1::lacZ promoter-reporter fusion construct showed a significant increase in β-galactosidase activity when expressing *Bm*HRG-1 (Fig. 2*E*). Based on both the ferrireductase and β-galactosidase activity assays, *Bm*HRG-1 is at least as effective as *Ce*HRG-1 or -4 at transporting heme.

### *Bm*HRGs promote heme uptake and increase the heme content of *B. malayi*

Immunoblot analysis of protein lysates (30 μg total protein extract) from *B. malayi* mf, adult females and males treated with increasing concentrations of heme revealed that endogenous protein levels of *Bm*HRG-1 were much higher in mf than adult females and almost undetectable in adult males (data not shown), suggesting that heme requirements may be more significant in adult females and the microfilarial stage. As with overexpression of HRGs in yeast, multiple oligomeric forms of *Bm*HRG-1 are detected within *B. malayi*. Moreover, the monomeric form detected by immunoblot analysis is slightly larger than the predicted monomer molecular mass (∼17 kDa), possibly suggesting the existence of *in vivo* posttranslational modifications.

**Figure 3.**
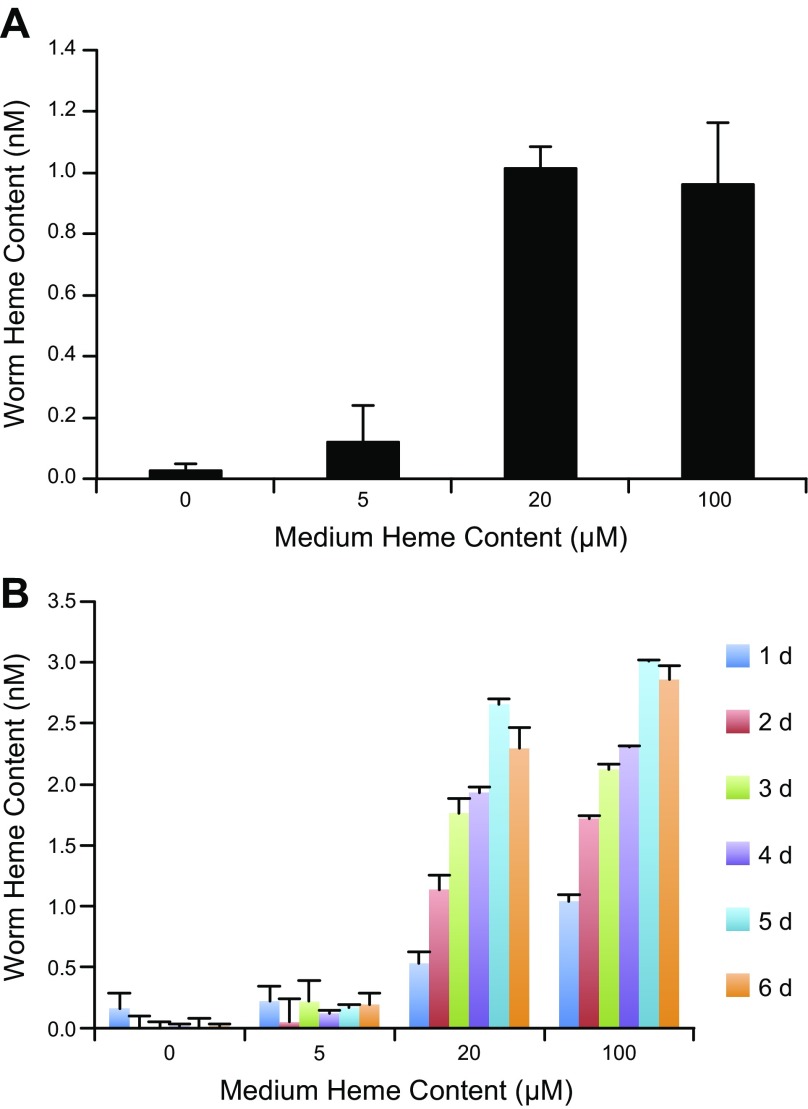
*Bm*HRG-1 and its effects on heme content in *B. malayi*. *A*) Heme content of 18 adult female worms after 7 d in culture with increasing concentrations of heme. *B*) Heme content of mf incubated in increasing concentrations of heme over time (6 d). Heme content of mf was normalized by the number of mf in each sample.

To evaluate the functionality of *Bm*HRGs in nematodes, the effect of exogenous heme on the motility of adult *B. malayi* males and females was examined. Most likely because of the cytotoxic effects of heme, worm motility was reduced by exposure to heme as compared to the untreated controls in both adult *B. malayi* females and males (Supplemental Fig. S1*A*, *B*). In addition, elevated concentrations of total heme were observed in *B. malayi* adult females and mf after being cultured in the presence of increasing concentrations of heme (**Fig. 3**). However, no significant differences in total worm heme content were observed above 20 μM heme exposure, suggesting total heme content is regulated *in vivo*. Heme concentrations were undetectable from a pool of 27 adult male worms, likely either because of a lack of biomass or limited heme uptake in this life cycle stage.

**Figure 4.**
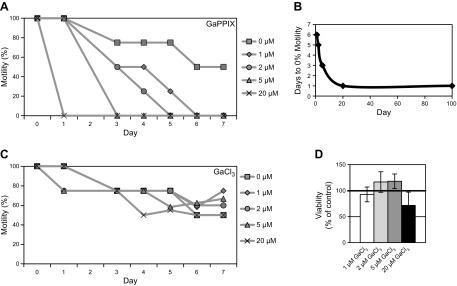
Effects of the heme analog GaPPIX on *B. malayi* motility. *A*) Motility of *B. malayi* females cultured with 0, 1, 2, 5, or 20 μM GaPPIX. *B*) Female *B. malayi* immotility as a function of GaPPIX concentration. *C*) Motility of *B. malayi* females cultured with 0, 1, 2, 5, or 20 μM GaCl_3_. Motility was assessed on a scale from 0 (nonmotile) to 4 (highly motile). Each data point represents motility from a single experiment where worms were scored as a group (6 adult worms/treatment group) (*A*, *C*). *D*) Viability of *B. malayi* females cultured in the presence of GaCl_3_ for 7 d. Viability was measured by the previously described MTT assay and the value of the control group (0 μM GaCl_3_) was set to 100 for comparison.

Highly cytotoxic noniron metalloporphyrins commonly exploit heme transport machinery and serve as potent antimicrobials (39). Studies have shown that *Ce*HRG-1 is capable of mediating the transport of 6 different noniron metalloporphyrins (6, 11). The functionality of *Bm*HRG-1 was further demonstrated by using the structurally similar heme analogs, GaPPIX and ZnMP. Gallium effectively mimics iron because of its similar atomic radius, electronic configuration, and +3 oxidation state (40). However, unlike Fe^3+^, Ga^3+^ cannot be reduced to Ga^2+^ under normal physiologic conditions (40), making it highly cytotoxic. Therefore, as expected, GaPPIX has been determined to be more than 800-fold more cytotoxic to *C. elegans* than is iron-containing heme (6). To determine whether GaPPIX may be taken up *via* the same mechanisms as heme in *B. malayi* and have similar cytotoxic effects, we exposed *B. malayi* adult females, *ex vivo*, to increasing concentrations of GaPPIX and monitored effects on motility. Exposure to GaPPIX had a much more pronounced negative effect on adult female *B. malayi* motility than heme (**Fig. 4*A***). Exposure to only a 5 μM concentration of GaPPIX caused adult worms (both male and female) to become immotile within 3 d in culture, whereas adult female *B. malayi* cultured in the same concentration of heme (5 μM) displayed ∼90% of the motility of control worms on d 3 (Supplemental Fig. S1*A*). Moreover, concentrations of GaPPIX as low as 1 μM eliminated worm motility after 6 d in culture (Fig. 4*B*). Neither motility nor viability (as measured by the MTT assay) of *B. malayi* adult females was significantly affected by exposure to the gallium salt, GaCl_3_ (Fig. 4*C*, *D*), suggesting the cytotoxic effect observed with GaPPIX was not because of spontaneous release of free gallium.

To further investigate heme uptake in *B. malayi*, live adult worms were soaked in ZnMP, a fluorescent heme analog. Fluorescence microscopy revealed concentrations as low as 5 μM ZnMP for 18 h resulted in detectable fluorescence (data not shown). Given the diffuse fluorescent signal of ZnMP, it is unclear what tissues or organs within the worm accumulate the heme analog. Concentrations of 40 μM ZnMP were used for further experiments, because this was the lowest concentration tested that produced the most consistent fluorescent accumulation.

**Figure 5.**
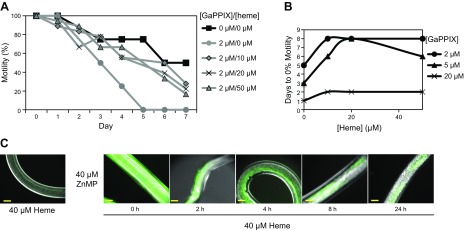
Characterization of heme uptake in *B. malayi.* *A*) *B. malayi* females were cultured in RPMI-1640 medium containing 2 μM GaPPIX and increasing hemin (0, 10, 20, or 50 μM). Motility was assessed as described in Materials and Methods on a scale from 0 (nonmotile) to 4 (highly motile). Each data point represents motility from a single experiment where worms were scored as a group (6 adult worms/treatment group) (*A*, *B*). The control groups (negative control: 0 μM GaPPIX/0 μM heme; positive control: 2 μM GaPPIX/0 μM heme) are taken from Fig. 4*A* as a reference. *B*) GaPPIX-induced immotility in female *B. malayi* is attenuated in the presence of heme. *B. malayi* females (6 worms/treatment group) were cultured in RPMI-1640 medium containing 2, 5, or 20 μM GaPPIX and increasing hemin (0, 10, 20, or 50 μM). *C*) Fluorescent labeling of female *B. malayi* worms incubated with 40 μM ZnMP for 18 h followed by a chase with 40 μM heme (hemin chloride). Worms were analyzed by epifluorescence microscopy at the indicated time points throughout the chase period (6 worms/time point) Scale bars, 100 μm. Representative images are shown.

To correlate the effects observed with the various heme analogs with heme transport, adult *B. malayi* females were exposed to 2 μM GaPPIX in the presence of increasing concentrations of heme (0, 10, 20, or 50 μM). The presence of heme in the medium provided protection from the GaPPIX-induced cytotoxicity, as indicated by the fact that worms incubated with GaPPIX + heme maintained ∼20% motility even after 7 d, whereas worms treated with only 2 μM GaPPIX were reduced to 0% motility after only 5 d in culture (**Fig. 5*A***). Furthermore, this heme-induced protection was evident even at higher concentrations of the toxic GaPPIX (5 and 20 μM) (Fig. 5*B*). To correlate ZnMP fluorescence with heme transport, the competitive effect of heme on ZnMP fluorescence was determined in a pulse–chase analysis. Worms were first fluorescently labeled by incubation in 40 μM ZnMP for 18 h before being washed to remove nonspecifically bound ZnMP and then incubated in 40 μM heme. The ZnMP fluorescence accumulated during the pulse slowly diminished over time in worms incubated in heme (Fig. 5*C*). Experiments performed in the absence of any unlabeled heme in the chase periods (to test for nonspecific fluorescence loss) showed no significant depreciation in ZnMP fluorescence in the 24-h time period (data not shown). Likewise, negligible fluorescence was detected in worms incubated only in 40 μM heme (Fig. 5C).

### *Bm*HRG-1 *in vitro* knockdown *via* hsiRNA

**Figure 6.**
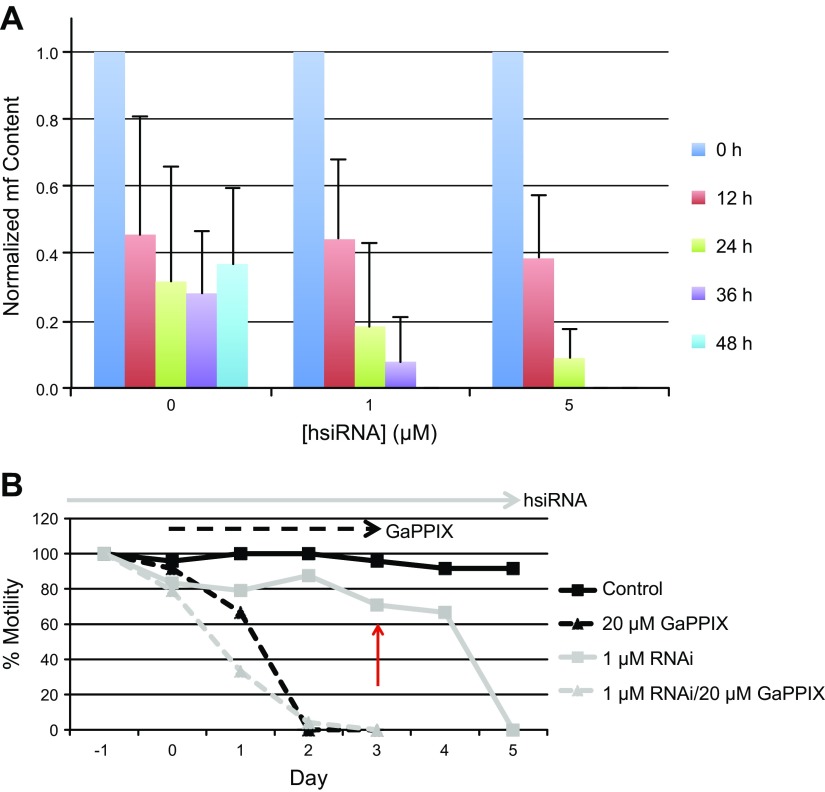
Knockdown of *Bm*HRG-1 by hsiRNA soaking. *A*) *B. malayi* females were cultured in RPMI-1640 medium containing increasing concentrations of *Bm*HRG-1 hsiRNA (0, 1, or 5 μM). Medium was changed every 12 h, at which point mf output for each treatment group was determined. Each hsiRNA treatment group mf count is normalized based on the 0-h timepoint. *B*) *B. malayi* females (6 worms/treatment group) were pretreated with or without 1 μM *Bm*HRG-1 hsiRNA for 24 h (d −1 to 0) before the addition of 20 μM GaPPIX (d 0). Motility was assessed as described in Materials and Methods on a scale from 0 (nonmotile) to 4 (highly motile). Each data point represents motility from a single experiment where worms were scored as a group (6 adult worms/treatment group). At d 3, 20 μM GaPPIX was added to the RNAi-only treatment group (red arrow) to determine if longer pretreatment with *Bm*HRG-1 hsiRNA would provide significant protection from GaPPIX cytotoxicity.

To investigate the role of *Bm*HRG-1 *in vitro*, adult *B. malayi* females were soaked in hsiRNA designed to target the *Bm*HRG-1 transcript to knockdown endogenous expression. Although motility of the worms was slightly affected (data not shown), mf production was severely affected by *Bm*HRG-1 knockdown (**Fig. 6*A***). Females treated with 1 and 5 μM *Bm*HRG-1 RNAi ceased mf production within 48 and 36 h, respectively. This is in stark contrast to control worms treated with no hsiRNA that were still readily producing mf after 2 d in culture. *Bm*HRG-1 knockdown was assessed using various experimental methods. Presoaking worms in 0.5 μM *Bm*HRG-1 hsiRNA followed by exposure to GaPPIX (0, 1, 2, 5, or 20 μM ± RNAi) provided a slight improvement in worm motility within the first 2 d (Supplemental Fig. S2). However, the GaPPIX-induced decline in worm motility was unaffected by the presence of *Bm*HRG-1 RNAi after d 2. Increasing the concentration of hsiRNA to 1 μM did not produce this same protective effect as the lower RNAi concentration and actually exacerbated the GaPPIX-induced cytotoxicity (Fig. 6*B*). Moreover, pretreatment with *Bm*HRG-1 hsiRNA for 4 d before exposure to GaPPIX did not provide any substantial protection from GaPPIX toxicity. In addition, presoaking adult female *B. malayi* with *Bm*HRG-1 hsiRNA had a concentration-dependent effect on ZnMP fluorescence (Supplemental Fig. S3*A*), whereas heme accumulation was seemingly unaffected (Supplemental Fig. S3*B*). Taken together, these results suggest that, although knockdown of *Bm*HRG-1 has an effect on mf production in adult females *in vivo*, other compensatory mechanisms (potentially other as of yet unidentified *Bm*HRGs) transport heme and heme analogs.

### Heme responsiveness of HRGs

**Figure 7.**
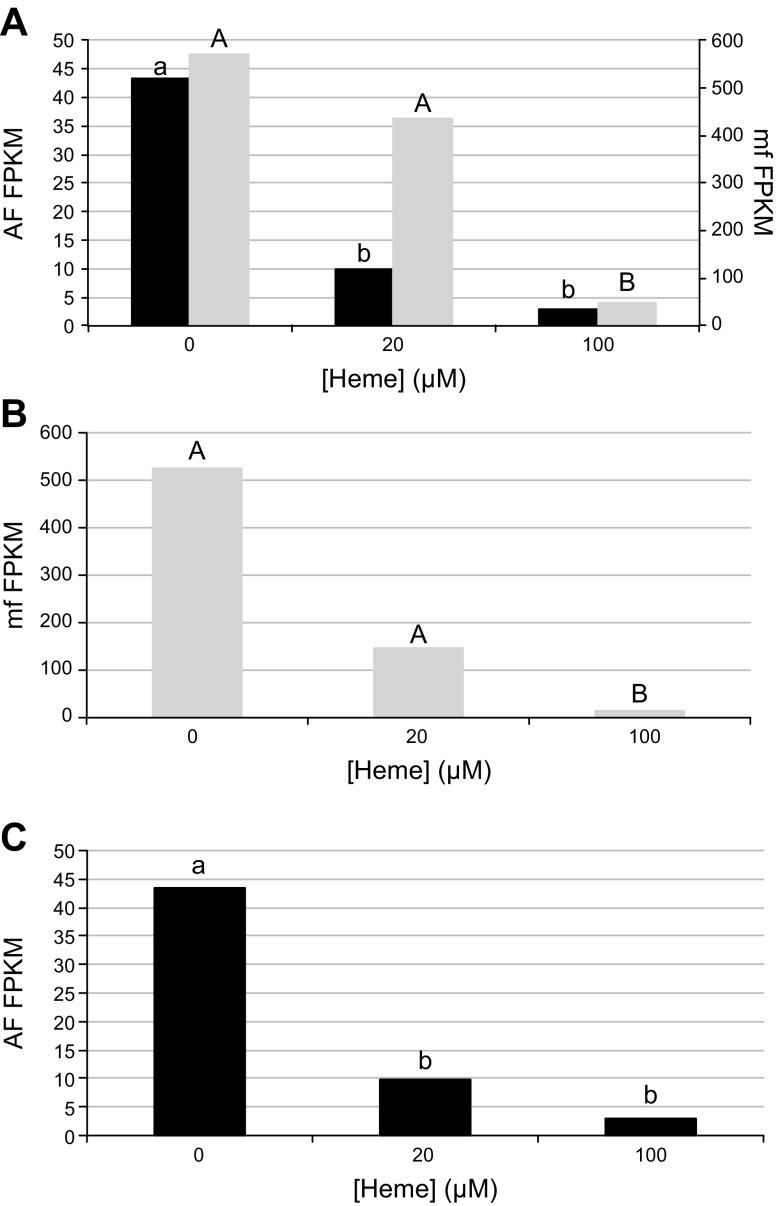
HRG expression in *B. malayi* adult females (AF) and mf in response to heme. *A*) FPKM values show expression of *Bm*HRG-1 is significantly down-regulated (*q* ≤ 0.01) in the presence of heme in *B. malayi* adult females (black bars) and mf (gray bars). At every heme concentration tested, *Bm*HRG-1 expression is significantly higher (*q* ≤ 0.01) in mf (gray bars, uppercase letters) than female worms (black bars, lowercase letters) (note difference in *y*-axis scales). *B*) *Bm*HRG-2 expression is significantly down-regulated (*q* ≤ 0.01) in mf at high (100 μM) heme concentrations. *C*) *Bm*MRP-5 expression is significantly down-regulated (*q* ≤ 0.01) in adult female *B. malayi* at 20 μM heme and above.

To assess whether the currently annotated *B. malayi* HRGs were indeed heme responsive, we performed RNA-sequencing on *B. malayi* that were exposed to various concentrations of exogenous heme (0, 20, or 100 μM) for 24 h. *Bm*HRG-1 expression is up-regulated in the mf stages, as compared to the adult stages (**Fig. 7*A***). Based on fragments per kilobase of transcript per million mapped reads (FPKM) values, *Bm*HRG-1 is down-regulated in a dose-dependent manner in both adult females (at heme concentrations as low 20 μM, black bars) and mf (at heme concentrations greater than 20 μM, gray bars). *Bm*HRG-1 was expressed in adult males at all heme concentrations tested; however, no significant differences were observed (data not shown).

Similar to *Bm*HRG-1 expression, *Bm*HRG-2 was significantly up-regulated in the mf compared to adults at every heme concentration examined (data not shown). *Bm*HRG-2 was significantly down-regulated in mf at high heme concentrations (100 μM; Fig. 7*B*), however, no significant differences were seen at any heme concentration for *Bm*HRG-2 in adult males or females.

Although up-regulated by low heme in *C. elegans* (13), the only significant difference in *Bm*MRP-5 was observed in adult females, in which *Bm*MRP-5 was significantly down-regulated at heme concentrations of 20 μM or above (Fig. 7*C*). No significant differences in *Bm*MRP-5 expression were observed between the different life cycle stages, except at 100 μM heme, where *Bm*MRP-5 was significantly up-regulated in mf in comparison to adult *B. malayi* (data not shown).

### *Bm*HRG-1 expression in adult females

**Figure 8.**
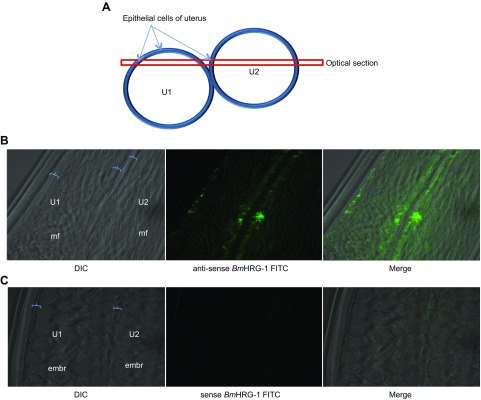
*Bm*HRG-1 expression by whole-mount *in situ* hybridization. *A*) Cross-section of *B. malayi* adult female showing 2 uterine branches (U1 and U2) containing intrauterine stages [mf or embryos (embr)]. *B*) Expression of *Bm*HRG-1 by whole-mount *in situ* hybridization with anti-sense FITC-labeled probe 120 d after infection. *C*) *Bm*HRG-1 sense probe image indicates negligible background staining.

Transcriptomic studies in both *B. malayi* and *D. immitis* indicate that heme biosynthesis genes are up-regulated in the microfilarial stage as well as the female body wall, intestine, and uterus (ref. 41 and unpublished results). Whole-mount *in situ* hybridization of adult *B. malayi* females revealed that *Bm*HRG-1 is indeed expressed in intrauterine mf, with the greatest expression observed in the epithelial cells of the uterine wall (**Fig. 8**).

## DISCUSSION

Originally identified because of its similarity to *Ce*HRG-1 (∼39%), *Bm*HRG-1 (*Bm*5182, WormBase ID: WBGene00225443) is slightly smaller than its *C. elegans* homolog, but maintains all the proven functional residues and motifs to make it an efficient transporter of heme. Although *Bm*HRG-1 displays sequence similarity to other *C. elegans* HRGs [*Ce*HRG-4 (29%), *Ce*HRG-5 (27%), and *Ce*HRG-6 (27%)], these other HRGs have not yet been identified in *B. malayi*. Presumably because of the lack of the C-terminal sorting motif that is present in *Ce*HRG-1, *Bm*HRG-1 expressed in yeast appears to localize, not only to the endocytic compartments, as *Ce*HRG-1 does, but also to the plasma membrane, as does *Ce*HRG-4. As with other HRGs, immunoblot analysis indicates *Bm*HRG-1 (expressed in yeast and from worm extracts) migrates as a monomer, as well as larger oligomeric forms. The monomeric form of endogenous *Bm*HRG-1 (from *B. malayi* extracts) is slightly larger than the expected 17 kDa, suggesting that it may be posttranslationally modified *in vivo*. A potential N-linked glycosylation site is present at position 34 of *Bm*HRG-1 (Asn34 in the E1 loop) and may account for the discrepancy in electrophoretic mobility between the yeast- and worm-derived extracts. Based on the RNAi-induced knockdown experiments, other heme transporters (*Bm*HRG-4, -5, or -6) are likely encoded within the *B. malayi* genome, yet remain unidentified.

It is evident that various stages of *B. malayi* (mf and adult males and females) can indeed acquire heme and heme analogs *via* specific heme transporters. Furthermore, both heme uptake assays in live worms and immunoblot analysis of whole worm extracts suggest protein levels and functional heme transport *via Bm*HRG-1 may be most critical during the mf stage. In agreement with our findings, transcriptomic studies of the *B. malayi* life cycle found that mature mf displayed higher expression levels of *Bm*HRG-1 than adults (42, 43). Previously, the highest levels of transcription for *Bm*HRG-1 were observed in the larval stage 3 (L3) (which we did not test in this study); however, the exact level of transcription at this stage was relatively uncertain, given the large variation in RPKM mapped read values (42, 43). In addition, the recent transcriptomic study of the life cycle of the related filarial nematode, *Dirofilaria immitis*, found that the 2 potential *Di*HRGs (nDi.2.2.2.g03420 and nDi.2.2.2.g07804) are both most highly expressed in the mf stage compared to L3 and L4, as well as adult males and females (44). Furthermore, gene ontology (GO) term analysis revealed that tetrapyrrole/heme binding functions were overrepresented in mf-associated transcripts compared to the other life cycle stages (44). The suggestion that heme transporters are highly expressed during the mf stage is particularly interesting, given that *B. malayi* mf typically survive for long periods in the blood of the mammalian host, where heme is plentiful. In contrast, adults are generally found feeding on the lymphatic fluid which typically contains only 32% as much heme (globulin content) as blood (45). Why heme transporters would be prevalent when the nematode is essentially bathed in heme remains unanswered. However, it may be related to heme availability, or lack thereof, in the insect vector stages of *B. malayi* development. *Plasmodium spp.* readily synthesizes heme despite the ability to acquire heme from hemoglobin during blood stages of infection (46). However, the same investigation found that the capability of synthesizing heme is critical for malarial parasite development in the liver and mosquito stages, suggesting that heme availability may be limiting within the mosquito. An analogous situation may exist for *B. malayi* development: mf may be accumulating stores of heme from the blood before mosquito-induced heme deprivation where the nematode may be forced to rely more on heme biosynthesis from *Wolbachia*.

*Ce*HRG-1 and -4 are expressed primarily in the intestine of the worm (6, 11). Like *Ce*HRG-1 and *Ce*MRP-5, *Bm*HRG-1 has a potential heme-responsive element (CGACATGTGATGAACTAATAATC) located 163 bp upstream of the transcriptional start site; however, in *B. malayi* it lacks critical elements required for intestinal expression (47). In addition, the intestine of *B. malayi* is relatively poorly developed or completely lacking (in the mf stage, L2, and L3) (48) and is thought to play little if any role in nutrient absorption (49). It is clear that many other nutrients (including leucine, adenosine and d-glucose) are selectively transported across the cuticle in adult and infective L3 *B. malayi* (50). Therefore, given the lack of a distinctly formed intestinal tract in *B. malayi* mf and that other nutrients are clearly obtained *via* transcuticular absorption, it seems plausible that heme uptake *via* HRGs may occur through the body wall and not the intestine.

Another question that arises is the availability of free unbound heme in any of the microenvironments experienced by *B. malayi* throughout its life cycle (human lymph: adult/L4; human blood: mf/infective L3; mosquito thoracic muscle cells and hemolymph: L1–L3). Estimates suggest the human body contains ∼3.5 g of iron, approximately 70% of which is contained as heme (51, 52), making it a vast iron resource. However, most heme in the blood is contained within red blood cells (essentially membranous sacs full of hemoglobin). Extracellular free heme, rarely found in the body because of its ability to induce the formation of radical oxygen species, is normally bound to hemopexin. However, serum hemoglobin levels (presumably bound to haptoglobin in a 2:1 hemoglobin:haptoglobin complex) are estimated to be anywhere between 80–800 nM (53). Various mechanisms exist in pathogenic species to capture and liberate intact heme and iron from host proteins (54); however, exactly how heme is liberated (possibly from free serum hemoglobin-haptoglobin complexes) before transport by *Bm*HRG-1 *in vivo* remains unclear. Although receptors specific for hemoglobin-haptoglobin and heme-hemopexin complexes are present in humans (CD163 and CD91) (55), such receptors involved in heme uptake (if present) could be potential filarial drug target candidates, but as of yet have not been identified in these nematodes.

Although many pathogenic bacterial species encode homologs of mammalian heme oxygenases that cleave the porphyrin ring of heme to liberate the iron (54), no heme oxygenase, which would serve to detoxify the peroxidase activity of excessive heme, as well as liberate iron for other uses (formation of Fe-S clusters), has been identified in nematodes. Studies in *C. elegans* have found that iron-deprived worms were unable to grow in the presence of normally adequate heme concentrations and were rescued only by increasing heme concentrations in the growth medium (6), suggesting that although heme is taken up and incorporated into hemoproteins, very little heme is broken down and utilized as a free iron source. Recent discoveries of not only a heme transporter, but a ferrous iron transporter and ferric iron reductase in *Leishmania* (56), reinforce the possibility that iron, as well as heme, may be transported and utilized within other parasites, such as *B. malayi*.

The acquisition of exogenous heme is critical for the survival of most nematodes, including *C. elegans*, which lack the ability to synthesize the essential cofactor. Filarial nematodes, such as *B. malayi* that contain the *Wolbachia* endosymbiont, may not be exclusive heme auxotrophs, but may also procure heme synthesized by their symbiont. Although little is known about iron metabolism in *wBm*, insect *Wolbachia* have been shown to be sensitive and responsive to host iron content (57).

The complementarity of the heme uptake–heme synthesis relationship of *B. malayi* and *Wolbachia* remains unclear. Genomic sequencing has revealed that *Wolbachia* contain the full repertoire of heme biosynthesis genes (8), whereas the host nematode does not (58). Biochemical studies suggest the *Wolbachia*-encoded heme biosynthetic pathway is essential for worm development and survival (59). Further, *Wolbachia* expression studies suggest heme regulates the encoded heme biosynthesis genes (unpublished results), yet *B. malayi* has the functional genes for heme uptake and distribution, as well as the laterally transferred functional gene for the last biochemical step (ferrochelatase). Transcriptomic studies in both *B. malayi* and *D. immitis* indicate that *Wolbachia* heme biosynthesis genes are up-regulated in male and female body wall, intestine, uterus, and testis, along with *Wolbachia* secretion systems (Sec and type IV secretion system components) (unpublished results). Taken together, the data lead us to speculate that *Wolbachia* helps supply heme to *B. malayi* for 2 potential purposes: *1*) for fertility (oogenesis) and other major heme requirements and/or *2*) for worm survival in the mosquito host component of the life cycle (mf stage through infective L3), where environmental heme is likely to be negligible or difficult to obtain. Heme biosynthesis remains a viable antifilarial target, but, as such, is likely not the only biochemical process involved in the mutualistic symbiosis.

Further investigations are needed, to better understand the complicated interactions between nematode and symbiont. What about other filarial nematodes that do not contain *Wolbachia?* Do they use only the one mechanism, that of heme uptake? It will be of interest to see how *B. malayi* worms cured of their *Wolbachia* endosymbionts (through tetracycline treatments) or filarial worms lacking *Wolbachia* (such as *Acanthocheilonema viteae*) react to exogenous heme.

## Abbreviations

ALA: δ-aminolevulinic acid
ALAS: δ-aminolevulinic acid synthase
*Bm*HRG-1–6: *Brugia malayi* heme-responsive gene 1–6
BmMRP-5: *Brugia malayi* multidrug resistance protein 5
*Ce*HRG-1–6: *Caenorhabditis elegans* heme-responsive gene 1–6
*Ce*MRP-5: *Caenorhabditis elegans* multidrug resistance protein 5
DIC: differential interference contrast
FPKM: fragments per kilobase of transcript per million mapped reads
GaPPIX: gallium protoporphyrin IX
HRG: heme-responsive gene
hsi: hairpin small interfering
L1–4: larval stage 1–4
mf: microfilariae
RNAi: RNA interference
RPKM: reads per kilobase of transcript per million mapped reads
SC: synthetic complete
SSC: saline sodium citrate
*wBm*: *Wolbachia* from *Brugia malayi*
ZnMP: zinc mesoporphyrin
